# Shortening intradermal rabies post-exposure prophylaxis regimens to 1 week: Results from a phase III clinical trial in children, adolescents and adults

**DOI:** 10.1371/journal.pntd.0006340

**Published:** 2018-06-06

**Authors:** Phirangkul Kerdpanich, Pornthep Chanthavanich, Mari Rose De Los Reyes, Jodor Lim, Delia Yu, Ma. Cecilia Ama, Zenaida Mojares, Daniela Casula, Ashwani Kumar Arora, Michele Pellegrini

**Affiliations:** 1 Department of Pediatrics, Division of Infectious Diseases, Phramongkutklao Hospital, Bangkok, Thailand; 2 Department of Tropical Pediatrics, Faculty of Tropical Medicine, Mahidol University, Bangkok, Thailand; 3 Research Institute for Tropical Medicine, Filinvest Corporate City, Alabang Muntinlupa City, the Philippines; 4 Asian Hospital and Medical Center, Filinvest Corporate City, Alabang Muntinlupa City, the Philippines; 5 De La Salle Health Sciences Institute-Congressional Road, Dasmarinas City, Cavite, the Philippines; 6 GSK, Siena, Italy; Universidad Nacional Mayor de San Marcos, PERU

## Abstract

**Background:**

This phase III clinical trial compared the immunogenicity and safety of a purified chick-embryo cell rabies vaccine (PCECV) administered according to a shortened post-exposure prophylaxis (PEP) 4-site/1-week intradermal regimen, compared with the currently recommended 2-site/Thai Red Cross (TRC) regimen.

**Methodology/Principal findings:**

This controlled, open-label, multi-center study (NCT02177032) enrolled healthy individuals ≥1 year of age, randomized into 4 groups to receive intradermal PCECV according to one of the 2 regimens, with or without human rabies immunoglobulin (HRIG) administration at first visit (in adults only). Rabies virus neutralizing antibody (RVNA) concentrations and percentages of participants with RVNA concentrations ≥0.5 IU/mL (considered as adequate concentrations following PEP) were assessed up to day (D) 365 post-first vaccination. Non-inferiority of the 4-site/1-week regimen to the 2-site/TRC regimen was demonstrated if at D49, the lower limit of the 95% confidence interval (CI) for the difference between groups in the percentage of participants with adequate RVNA concentrations was >-5%. Of the 443 participants receiving the 4-site/1-week regimen, 88 adults received HRIG; 442 participants received the 2-site/TRC regimen (88 with HRIG). All participants achieved adequate RVNA concentrations by D14. At D49, the difference in percentage of participants with adequate RVNA concentrations between the 4-site/1-week and the 2-site/TRC groups was -1 (95%CI: -2.4–0.0); thus, non-inferiority was concluded. RVNA geometric mean concentrations were 18 IU/mL in 4-site/1-week groups and 12 IU/mL in 2-site/TRC groups at D14, and subsequently declined in all groups. RVNA concentrations were consistently lower in adults with HRIG administration than in those without. The 2 regimens had similar safety profiles. Of the 15 serious adverse events reported in 4-site/1-week groups and 19 in 2-site/TRC groups, none were vaccination-related.

**Significance:**

The data suggest that the 4-site/1-week regimen might be an alternative to current recommendations, with potential benefits in terms of improved cost-efficiency and compliance to vaccination.

## Author summary

Rabies is a deadly, but vaccine-preventable disease which still causes tens of thousands of deaths yearly, mostly in Asia and Africa. Rabies virus is spread via the saliva of infected mammals to humans, usually through bites or contamination of open wounds. Access to measures like wound cleansing with soap and rabies vaccination immediately after contact with a suspected rabid animal (exposure) can be life-saving. The post-exposure vaccination schedule currently recommended by the World Health Organization for intradermal injection is the Thai Red Cross regimen, requiring 4 clinic visits in one month, with 2 injections given at each visit on days (D) 0 (day of the contact), 3, 7, and 28. In this study, we evaluated the antibody responses and the safety profile of a new shortened schedule, requiring 3 clinic visits and only 1 week to complete, consisting of 4 intradermal injections given at each visit on D0, 3, and 7 (the 4-site/1-week regimen). The study was conducted in the Philippines and Thailand which enrolled 885 healthy volunteers, at least 1 year of age, with no real exposure to rabies. The two schedules induced adequate antibody responses in similar proportion of volunteers at day 49. The vaccine administration according to both schedules was well tolerated.

## Introduction

Rabies is an acute viral disease, caused by viruses belonging to the *Lyssavirus genus* of the *Rhabdoviridae* family [1]. Although rabies is almost eliminated in industrialized countries, it is still estimated to cause more than 60,000 deaths each year worldwide, of which the vast majority occur in Asia and Africa [2]. Despite the fact that the disease is completely preventable and that recent massive campaigns targeting its elimination were launched in endemic regions [3], rabies continues to be listed as a neglected tropical disease by the World Health Organization (WHO) [4].

Prevention of rabies by post-exposure prophylaxis (PEP), including vaccination, is highly effective when administered promptly after suspected exposure. Current recommendations also indicate concomitant administration of rabies immunoglobulins (RIG) for WHO category III rabies exposures [5]. In endemic regions, intradermal (ID) vaccination regimens have proven to be more cost-effective than intramuscular (IM) ones and are therefore used preferentially. The recommended WHO ID regimen for PEP, the updated 2-site Thai Red Cross (2-site/TRC) regimen, takes approximately 1 month and requires 4 clinic visits with 2 doses of vaccine administered on each of the days (D) 0, 3, 7, and 28 [1].

However, to date, prevention of rabies remains inadequate in developing regions, where access to medical care is not optimal [6]. Reducing the number of required visits would reduce costs, while at the same time increasing compliance to the full PEP course. A newly proposed PEP regimen (not yet recommended by the WHO) allows completion of the ID immunization within 1 week through administration of 4 vaccine doses on D0, D3, and D7, and has shown encouraging results in clinical trials (4-site/1week) [7, 8].

This study aimed to demonstrate the non-inferiority of the immune response following vaccination with a purified chick embryo cell culture vaccine (PCECV) according to the new 4-site/1-week ID regimen compared to the updated conventional 2-site/TRC ID regimen with or without concomitant administration of human RIG (HRIG).

## Methods

### Study design and participants

This was a phase III, randomized, age-stratified, controlled, open-label study carried out in 4 centers in the Philippines and 2 centers in Thailand, between June 2014 and August 2015.

The study enrolled healthy individuals ≥1 year of age, who had never received rabies vaccines or RIG, had not previously been exposed to rabies, and had no history of allergy or contraindications to the study vaccine or HRIG components. Pregnant women were not enrolled in the study. The full list of exclusion criteria is presented in **S1 Text**.

**Fig 1:**
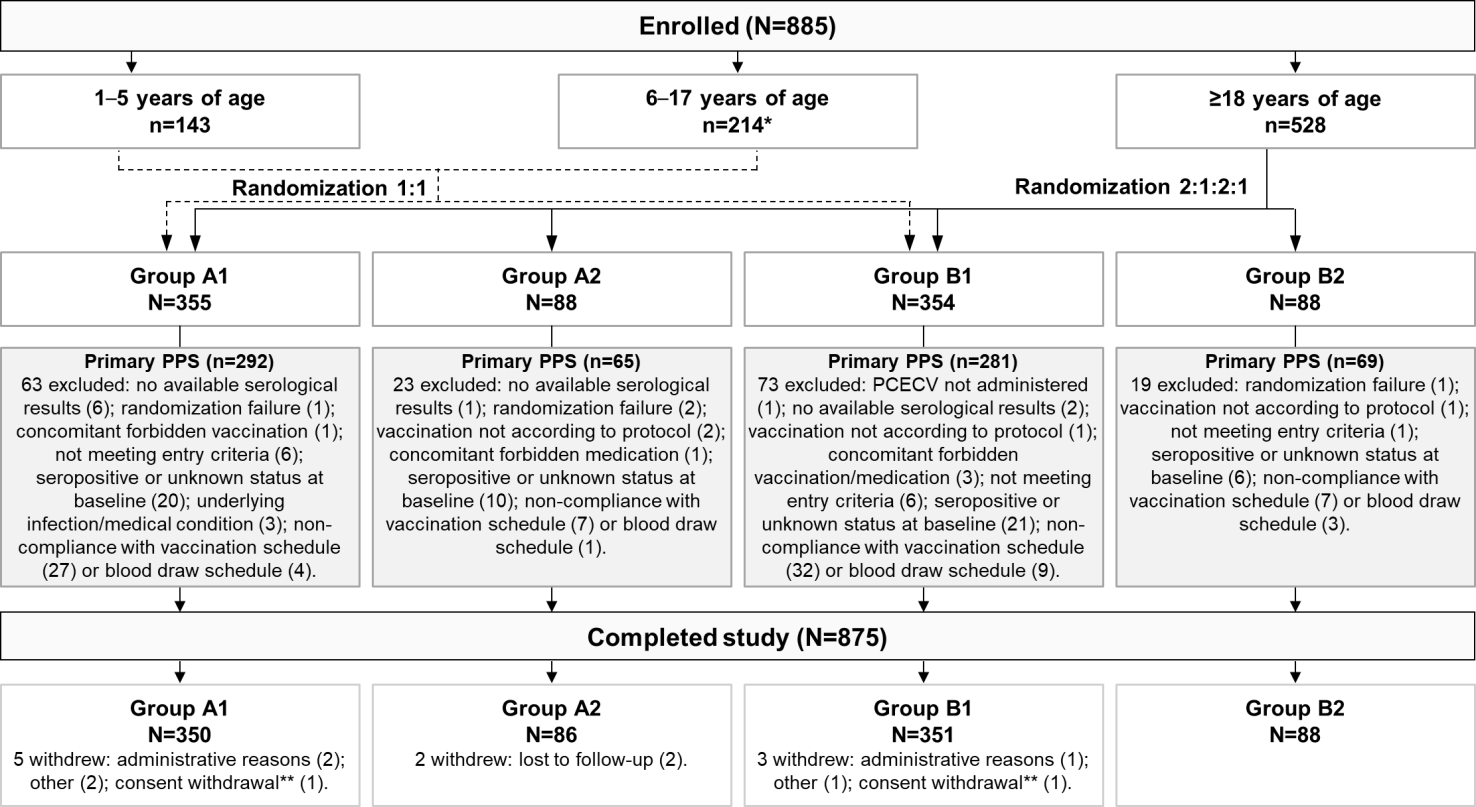
Participant flow diagram. Group A1, participants receiving PCECV according to the 4-site/1-week ID regimen; Group A2, participants receiving PCECV according to the 4-site/1-week ID regimen and HRIG at first visit; Group B1, participants receiving PCECV according to the 2-site/TRC regimen; Group B2, participants receiving PCECV according to the 2-site/TRC regimen and HRIG at first visit; PCECV, purified chick embryo cell culture vaccine; ID, intradermal; HRIG, human rabies immunoglobulin; TRC, Thai Red Cross; n, number of participants in each age stratum; N, number of participants in each group; PPS, per-protocol set. * One 11-year-old participant was erroneously randomized in Group B2. ** Not due to an adverse event.

Individuals were enrolled based on the following age strata: 1–5 years, 6–17 years, and ≥18 years. Children and adolescents younger than 18 years were randomly assigned (1:1) to one of the groups receiving PCECV according to the 4-site/1-week ID regimen (Group A1) or the 2-site/TRC regimen (Group B1). Adults were randomized (2:1:2:1) into 4 groups to receive PCECV according to the 4-site/1-week ID regimen alone (Group A1) or with concomitant HRIG administration at first visit (Group A2), or PCECV according to the 2-site/TRC ID regimen alone (Group B1) or with concomitant HRIG administration at first visit (Group B2) (**Fig 1; S2 Text**).

### Ethics statement

The participants or their parents/legally accepted representatives (LARs) were required to be able to understand and comply with all study procedures, and a written informed consent was obtained before study enrolment. The study was conducted in accordance with the Declaration of Helsinki and the principles of Good Clinical Practice. The study protocol, amendments, and informed consent forms from the participants or their parents/LARs were reviewed and approved by regional Independent Ethics Committees. The study is registered at ClinicalTrials.gov (NCT02177032). All patient-level data were anonymized.

### Study objectives and endpoints

The primary objective was to assess non-inferiority of the immune response of the 4-site/1-week ID PEP regimen to that of the currently recommended 2-site/TRC ID regimen of PCECV, with or without HRIG administration, as measured by the percentage of participants with rabies virus neutralizing antibody (RVNA) concentrations ≥0.5 international units (IU)/mL in the whole study population, at D49. Non-inferiority was demonstrated if the lower limit (LL) of the 2-sided 95% confidence interval (CI) around the difference (Group A1 + Group A2 minus Group B1 + Group B2) in the percentage of participants with RVNA concentrations ≥0.5 IU/mL was greater than -5%.

Non-inferiority of the immune response of the 4-site/1-week ID PEP regimen to that of the currently recommended 2-site/TRC regimen of PCECV, with or without HRIG administration, was also tested in terms of RVNA geometric mean concentrations (GMCs) in the whole study population, at D49. Non-inferiority was demonstrated if the LL of the 2-sided 95% CI around the GMC ratio (Group A1 + Group A2 over Group B1 + Group B2) was >0.667.

Considering the average incubation period for rabies (between 20 and 60 days) [9] and to allow the immunogenicity evaluation at approximately 3 weeks after last vaccine administration (D28 for Groups B1 and B2), D49 was selected as time point for the assessment of the non-inferiority endpoints.

Other secondary objectives compared the antibody responses at D7, D14, D90, D180, and D365, in terms of RVNA GMCs and percentages of participants with RVNA concentrations ≥0.5 IU/mL following each PCECV regimen, in the entire study population and in adults only, and in study groups with and without HRIG administration. The safety and tolerability of both PCECV regimens were also evaluated.

### Study vaccine

PCECV is a lyophilized vaccine produced from the Flury LEP-25 strain grown in primary cultures of chick fibroblasts and inactivated with β-propiolactone [10, 11]. Prior to administration, PCECV (commercial lot 2470, with a potency of 6.87 IU/mL) was reconstituted with sterile water for injection to yield a final volume of 1 mL.

**Fig 2:**
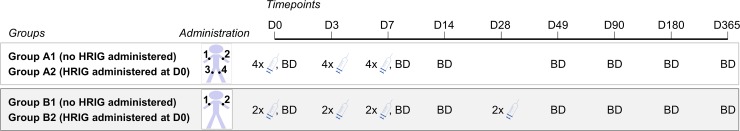
Study design. Group A1, participants receiving PCECV according to the 4-site/1-week ID regimen; Group A2, participants receiving PCECV according to the 4-site/1-week ID regimen and HRIG at first visit; Group B1, participants receiving PCECV according to the 2-site/TRC ID regimen; Group B2, participants receiving PCECV according to the 2-site/TRC ID regimen and HRIG at first visit; PCECV, purified chick embryo cell culture vaccine; ID, intradermal; HRIG, human rabies immunoglobulin; TRC, Thai Red Cross; D, day; BD, blood draw. Administration schemes represent injection sites. The syringe symbolizes 1 injection of 0.1 mL of PCECV vaccine.

At each vaccination visit (**Fig 2**), participants in Groups A1 and A2 received 4 injections of 0.1 mL of PCECV (one dose in each deltoid and anterolateral thigh area), while participants in Groups B1 and B2 received 2 injections of 0.1 mL of PCECV (one dose in each deltoid). All injections were administered via ID route.

A dose of 20 IU/kg body weight of commercially available HRIG (*Berirab*, Behring GmbH, lot number 08547142) was administered on D0, concomitantly with the vaccination, to all adults in Groups A2 and B2, by IM injection into the gluteal muscle. Because this was a simulated PEP study in healthy volunteers, the use of HRIGs was preferred to those of equine origin, in order to avoid exposing study participants to any potential (although usually rare) safety issues, linked to the use of material of heterologous origin.

### Immunogenicity assessment

Blood samples (approximately 5 mL) for immunogenicity testing were collected at D0, 7, 14, 49, 90, 180, and 365 (**Fig 2**). RVNA levels were assessed by the rapid fluorescent focus inhibition test [12], which was performed at the Kansas State University Rabies Laboratory, Manhattan, KS, United States [13]. RVNA concentrations ≥0.5 IU/mL by D14 are considered by the WHO as an adequate response to rabies PEP [1].

### Reactogenicity and safety assessment

After each vaccination, all participants were observed for at least 30 minutes and evaluated for adverse events (AEs). Participants or their parents/LARs recorded any AEs occurring after this interval on diary cards, which they returned at the next visit.

All solicited local and systemic AEs were recorded for 3 days (D0–2) following first vaccination visit, 4 days following second vaccination visit, and 7 days following third and fourth (for Groups B1 and B2 only) vaccination visits. Solicited local AEs were induration, swelling, erythema, and tenderness for children 1–5 years of age, and induration, swelling, erythema, and pain for participants older than 6 years. Solicited systemic AEs were change in eating habits, sleepiness, vomiting, diarrhea, and irritability in the 1–5 years age stratum, and generalized arthralgia, headache, fatigue, rash, vomiting, diarrhea, and loss of appetite in all other participants. Solicited AEs that were not resolved during the post-vaccination observation periods were also recorded as unsolicited AEs. The relationship between AEs and study treatment (“not related”, “possibly related”, and “probably related”) was determined by the investigators. Unsolicited AEs were followed for 29 and 50 days after each vaccination for participants in Groups A1 and A2 and Groups B1 and B2, respectively. Serious AEs (SAEs) and AEs leading to withdrawal were recorded throughout the study. A SAE was defined as any untoward medical occurrence that resulted in death, hospitalization or prolongation of hospitalization, persistent or significant disability/incapacity, congenital anomaly or birth defect, or was life-threatening.

### Statistical analyses

Immunogenicity assessments were carried out in the per-protocol sets (PPSs) at each time point, including all participants who complied with the vaccination schedule, had immunogenicity data at the relevant time points, and did not have major protocol deviations. The non-inferiority objectives were assessed in the primary PPS (PPS at D49).

The percentage of participants with RVNA concentrations ≥0.5 IU/mL and RVNA GMCs were tabulated with 95% CIs for each group, by time point and age stratum. Between-group differences in the percentage of participants with RVNA concentrations ≥0.5 IU/mL were calculated using a binomial distribution, and associated CIs were constructed using the Miettinen-Nurminen method [14]. The GMCs, the within-group geometric mean ratios, and the associated 2-sided 95% CIs for each group were computed by the exponentiation of the least square means of the logarithmically transformed concentrations (and their differences), and the 95% CIs were obtained from an analysis of variance with vaccine regimen, age stratum, and center as factors.

Safety assessments were performed in the safety set, which included participants who had received at least one dose of PCECV and had solicited or unsolicited AE data (i.e., any safety information) post-vaccination. The number and percentage of participants with solicited and unsolicited AEs, and SAEs were reported for each vaccine group and for each age stratum.

Analyses were performed using SAS (Statistical Analysis System) 9.1 software.

### Sample size and power

For the primary objective, the high lethality of rabies was taken into account to establish the non-inferiority margin. Based on previous studies, where the vast majority of participants achieved adequate RVNA concentrations after 4 PCECV doses [15], the percentages of participants with RVNA concentrations ≥0.5 IU/mL were assumed to reach 97% in Groups A1 and B1, 95% in Groups A2 and B2, and 96% in the whole population. Accounting for a non-evaluable/drop-out rate of 20%, non-inferiority could be shown with a power of 89.8% for a total of 876 enrolled participants (438 in Groups A1 and A2 and 438 in Groups B1 and B2).

Sample size and power estimations were computed using the Query Advisor 7.0 software.

## Results

### Demographics

Out of the 885 enrolled participants, all attended at least 1 visit, and 875 completed the study. Disposition of participants by groups, reasons for withdrawal from the study, and protocol deviations leading to elimination from the primary PPS are given in **Fig 1**.

The mean age was 22.9 years, 54% of participants were female and all were of Asian heritage. Study groups were balanced in terms of demographic and baseline characteristics, apart from age (due to the assignment of only adults in groups receiving HRIG) (**Table 1)**.

**Table 1:** Demographics characteristics of participants at enrolment.

	Group A1 (N = 355)	Group A2 (N = 88)	Group B1 (N = 354)	Group B2 (N = 88)	Total (N = 885)
Age (mean±SD), years	20.0±15.0	32.7±10.8	20.6±15.1	34.4±11.2	22.9±15.3
Participants in each age stratum, n (%)					
1–5 years	72 (20%)	0 (0%)	71 (20%)	0 (0%)	143 (16%)
6–17 years	106 (30%)	0 (0%)	107 (30%)	1 (1%)	214 (24%)
≥18 years	177 (50%)	88 (100%)	176 (50%)	87 (99%)	528 (60%)
Female gender, n (%)	202 (57%)	44 (50%)	179 (51%)	51 (58%)	476 (54%)
Asian heritage, n (%)	355 (100%)	88 (100%)	354 (100%)	88 (100%)	885 (100%)
Weight (mean±SD), kg	44.2±22.7	61.0±13.7	45.4±22.8	64.2±14.1	48.3±22.4
Height, (mean±SD), cm	140.0±26.4	160.3±9.3	141.0±27.5	160.0±8.0	144.4±25.6

Group A1, participants receiving PCECV according to the 4-site/1-week ID regimen; Group A2, participants receiving PCECV according to the 4-site/1-week ID regimen and HRIG at first visit; Group B1, participants receiving PCECV according to the 2-site/TRC ID regimen; Group B2, participants receiving PCECV according to the 2-site/TRC ID regimen and HRIG at first visit; PCECV, purified chick embryo cell culture vaccine; ID, intradermal; HRIG, human rabies immunoglobulin; TRC, Thai Red Cross; N, number of participants in each group; n (%), number (percentage) of participants in each category; SD, standard deviation.

Compliance with the vaccination schedule was high, with at least 98% of participants in Groups A1, A2 and B1 and all participants in Group B2 receiving all vaccine doses as planned.

### Immunogenicity

At D49, the percentages of participants with RVNA concentrations ≥0.5 IU/mL were 99% (95% CI: 98%–100%) in groups receiving the 4-site/1-week ID regimen (Group A1 + Group A2), and 100% (95% CI: 99%–100%) in groups receiving the 2-site/TRC ID regimen (Group B1 + Group B2). The LL of the 2-sided 95% CI on the between-group difference was -2.4% which is above the pre-specified non-inferiority margin. Thus, the primary objective was met.

In the whole population, for both vaccination regimens, all participants achieved RVNA concentrations ≥0.5 IU/mL by D14 and the vast majority of them (90% in Group A1 + Group A2 and 83% in Group B1 + Group B2), maintained adequate RVNA concentrations at 1 year (D365) after first vaccination (**Table 2**).

**Table 2:** Summary of between-groups comparison of immune response to different PCECV regimens in terms of percentage of participants with RVNA concentrations ≥0.5 IU/mL and GMCs (per-protocol set at each time point).

	Groups						Difference	GMC Ratio
	**A1+A2**			**B1+B2**			**(A1+A2)/(B1+B2)**	**(A1+A2)/(B1+B2)**
	N	% (95% CI)	GMC (95% CI)	N	% (95% CI)	GMC (95% CI)	% (95% CI)	value (95% CI)
D0	364	0 (0–1)	0.05 (0.05–0.05)	362	0 (0–1)	0.05 (0.05–0.05)	0 (-1.1–1)	1 (1–1)
D7	363	18 (15–23)	0.23 (0.21–0.25)	362	10 (7–13)	0.14 (0.13–0.15)	9 (3.8–13.9)	1.04 (0.84–1.29)
D14	357	100 (99–100)	18 (17–20)	361	100 (98–100)	12 (10–13)	0 (-1.0–1.6)	1.68 (1.35–2.10)
D49	357	99 (98–100)	4.58 (4.15–5.06)	350	100 (99–100)	10 (9.29–11)	-1 (-2.4–0)	0.46 (0.37–0.58)
D90	358	96 (94–98)	2.32 (2.09–2.56)	362	99 (98–100)	3.68 (3.32–4.07)	-3 (-6–-1.4))	0.68 (0.54–0.85)
D180	357	93 (90–95)	1.59 (1.41–1.78)	360	93 (90–96)	1.44 (1.28–1.62)	-1 (-4.5–3.2)	1.22 (0.94–1.59)
D365	356	90 (86–93)	1.59 (1.41–1.79)	352	83 (79–87)	1.14 (1.01–1.28)	6 (1.3–11.5)	1.67 (1.27–2.19)
	**A1**			**B1**			**A1-B1**	**A1/B1**
	N	% (95% CI)	GMC (95% CI)	N	% (95% CI)	GMC (95% CI)	% (95% CI)	value (95% CI)
D0	298	0 (0–1)	0.05 (0.05–0.05)	290	0 (0–1)	0.05 (0.05–0.05)	0 (-1.3–1.3)	1 (1–1)
D7	297	21 (16–26)	0.23 (0.21–0.25)	290	10 (7–14)	0.13 (0.12–0.14)	11 (5.5–17.1)	1.77 (1.56–2.02)
D14	292	100 (99–100)	19 (17–21)	289	100 (99–100)	12 (11–13)	0 (-1.3–1.3)	1.59 (1.39–1.82)
D49	292	100 (99–100)	5.04 (4.56–5.57)	281	100 (99–100)	10 (9.48–12)	0 (-1.3–1.4)	0.48 (0.42–0.55)
D90	293	99 (97–100)	2.54 (2.29–2.82)	290	100 (98–100)	3.8 (3.42–4.21)	-1 (-2.7–1.0)	0.67 (0.58–0.77)
D180	292	96 (93–98)	1.73 (1.53–1.95)	289	96 (92–98)	1.5 (1.32–1.69)	1 (-2.7–4.2)	1.16 (0.99–1.35)
D365	291	95 (92–97)	1.73 (1.53–1.95)	283	87 (82–90)	1.2 (1.06–1.36)	8 (3.6–13.2)	1.44 (1.23–1.7)
	**A2**			**A1 (adults only)**			**A2-A1 (adults only)**	**A2/A1 (adults only)**
	N	% (95% CI)	GMC (95% CI)	N	% (95% CI)	GMC (95% CI)	% (95% CI)	value (95% CI)
D0	66	0 (0–5)	0.05 (0.05–0.05)	151	0 (0–2)	0.05 (0.05–0.05)	0 (-2.5–5.5)	1 (1–1)
D7	66	8 (3–17)	0.21 (0.17–0.26)	150	19 (13–26)	0.15 (0.13–0.17)	-11 (-19.6–-1.0)	1.39 (1.10–1.76)
D14	65	100 (94–100)	13 (11–15)	146	100 (98–100)	15 (13–17)	0 (-5.6–2.6)	0.85 (0.68–1.08)
D49	65	95 (87–99)	1.97 (1.60–2.43)	146	100 (98–100)	4.47 (3.90–5.13)	-5 (-12.7–-1.6)	0.44 (0.34–0.57)
D90	65	83 (72–91)	1.01 (0.81–1.27)	149	98 (94–100)	2.28 (1.96–2.65)	-15 (-26–-7.1)	0.44 (0.34–0.58)
D180	65	77 (65–86)	0.88 (0.67–1.14)	146	94 (89–97)	1.92 (1.61–2.29)	-17 (-29–-7.1)	0.46 (0.33–0.63)
D365	65	66 (53–77)	0.68 (0.53–0.88)	145	91 (85–95)	1.42 (1.19–1.69)	-25 (-37.8–-13.2)	0.48 (0.35–0.66)
				**B2**			**A2-B2**	**A2/B2**
				N	% (95% CI)	GMC (95% CI)	% (95% CI)	value (95% CI)
D0				72	0 (0–5)	0.05 (0.05–0.05)	0 (-5.1–5.5)	1 (1–1)
D7				72	10 (4–19)	0.17 (0.14–0.21)	-2 (-12.3–8.2)	1.22 (0.93–1.6)
D14				72	99 (93–100)	8.37 (6.96–10)	1 (-4.3–7.5)	1.53 (1.17–1.99)
D49				69	100 (95–100)	6.37 (5.21–7.78)	-5 (-12.8–1.0)	0.31 (0.23–0.41)
D90				72	99 (93–100)	2.25 (1.81–2.79)	-16 (-26.7–-6.9)	0.45 (0.33–0.62)
D180				71	85 (74–92)	1.05 (0.81–1.35)	-8 (-21.2–5.8)	0.84 (0.58–1.21)
D365				69	70 (57–80)	0.59 (0.46–0.76)	-3 (-19.2–12.4)	1.15 (0.8–1.65)

Group A1, participants receiving PCECV according to the 4-site/1-week ID regimen; Group A2, participants receiving PCECV according to the 4-site/1-week ID regimen and HRIG at first visit; Group B1, participants receiving PCECV according to the 2-site/TRC ID regimen; Group B2, participants receiving PCECV according to the 2-site/TRC ID regimen and HRIG at first visit; PCECV, purified chick embryo cell culture vaccine; ID, intradermal; TRC, Thai Red Cross; CI, confidence interval; D, day; GMC, geometric mean concentration; HRIG, human rabies immunoglobulin; n (%), number (percentage) of participants with RVNA concentrations ≥0.5 IU/mL; N, number of participants with available results; RVNA, rabies virus neutralizing antibody.

RVNA GMCs peaked at D14 and declined at subsequent time points. At D49, the RVNA GMC ratio (Group A1 + Group A2 over Group B1 + Group B2) was 0.46 (95% CI: 0.37–0.58), and the LL of the 95% CI was below the pre-specified non-inferiority margin; thus, the secondary objective was not met.

At D365, a higher immune response was observed in the groups receiving the 4-site/1-week regimen than in those receiving the 2-site/TRC regimen, as shown by the between-group GMC ratios (**Table 2**).

In groups not receiving HRIG, all study participants achieved adequate RVNA levels by D14, and a higher proportion of participants in Group A1 (95%) than in Group B1 (87%) maintained adequate neutralizing antibody levels for 1 year following first vaccination (**Table 2**). In both groups, RVNA GMCs peaked at D14 and then declined up to D365, with higher values being observed in Group A1 than in Group B1 at D14 and D365 (**Table 2**).

At all-time points except D14, the percentage of adults with adequate RVNA concentrations was lower in Group A2 than in group A1 (**Table 2**). RVNA GMCs were consistently lower in Group A2 than in Group A1 (**Table 2**).

Comparable percentages of adults with RVNA concentrations ≥0.5 IU/mL were observed for groups A2 and B2 for all time points, except at D90 when the percentage of adults with adequate RVNA levels was significantly lower in Group A2 than in Group B2 (**Table 2**). RVNA GMCs peaked in both groups at D14, when a significantly higher GMC was observed in Group A2 than in Group B2. RVNA levels declined in both groups at subsequent time points (**Table 2**).

The same trend as described for the overall population was observed in the different age strata evaluated, including in the youngest participants (children 1–5 years of age).

### Reactogenicity and safety

At least 1 solicited AE was reported in 57% (Group A1) and 65% (Group A2) of participants receiving the 4-site/1 week regimen and in 59% (Group B1) and 62% (Group B2) of participants receiving the 2-site/TRC regimen (**Table 3**).

**Table 3:** Number and percentages of participants with solicited adverse events after any vaccination, and unsolicited and serious adverse events throughout the study (safety set).

	Group A1	Group A2	Group B1	Group B2
	N = 356	N = 85	N = 353	N = 89
Any solicited AE	202 (57%)	55 (65%)	208 (59%)	55 (62%)
Local	144 (40%)	35 (41%)	155 (44%)	33 (37%)
Systemic	119 (33%)	36 (42%)	118 (33%)	41 (46%)
Any unsolicited AE	260 (73%)	71 (84%)	280 (79%)	73 (82%)
Possibly or probably related unsolicited AEs	236 (66%)	71 (84%)	229 (65%)	71 (80%)
Any SAE	12 (3%)	3 (4%)	14 (4%)	5 (6%)
Possibly or probably related SAEs	0 (0%)	0 (0%)	0 (0%)	0 (0%)

Group A1, participants receiving PCECV according to the 4-site/1-week ID regimen; Group A2, participants receiving PCECV according to the 4-site/1-week ID regimen and HRIG at first visit; Group B1, participants receiving PCECV according to the 2-site/TRC ID regimen; Group B2, participants receiving PCECV according to the 2-site/TRC ID regimen and HRIG at first visit; PCECV, purified chick embryo cell culture vaccine; ID, intradermal; HRIG, human rabies immunoglobulin; TRC, Thai Red Cross; N, number of participants with available results; AE, adverse event; SAE, serious adverse event.

Solicited local reactions were more frequent in the 1–5 years age stratum (76% and 68% of children in Groups A1 and B1, respectively) than in the other age strata (31%–41% and 33%–41% of individuals receiving the 4-site/1-week ID regimen and the 2-site/TRC ID regimen, respectively; **S1 Table**). The most frequently reported solicited local AEs were injection site tenderness in the 1–5 years age stratum (ranging from 7% to 21% of children across study groups), injection site pain in the 6–17 years age stratum (in 4–14% of participants in all groups), and injection site erythema in the ≥18 years age stratum (ranging from 1% to 16% of adults across study groups).

Solicited systemic AEs were reported in 33%–42% of participants receiving the 4-site/1-week ID regimen and 33%–46% of participants receiving the 2-site/TRC ID regimen (**Table 3**) and were more frequently reported in the ≥18 years age stratum (**S1 Table**). After any vaccination, the most frequent solicited systemic AEs were fever (reported in 7% of children in Group A1 and 17% in Group B1) and sleepiness (in 14% and 13% of children in Groups A1 and B1, respectively) in the 1–5 years age stratum; and headache (in 13% of participants in Group A1 and 16% in Group B1) and fatigue (for 12% of participants in Group A1 and 11% in Group B1) in the 6–17 years age stratum. After any vaccination, headache (with incidences ranging from 19% in Group B1 to 35% in Group B2) and fatigue (ranging from 19% in Group A2 to 27% in Group B2) were also the most frequently reported solicited systemic AEs for participants aged ≥18.

Severe solicited local and systemic AEs were reported in 0%–2% of participants in all age groups; most solicited AEs were mild to moderate in intensity.

Unsolicited AEs were reported in 73% and 84% of participants in Groups A1 and A2, respectively, and in 79% and 82% of participants in Groups B1 and B2, respectively. Unsolicited AEs at least possibly or probably related to vaccination were reported in 66%, 84%, 65%, and 80% of participants in Groups A1, A2, B1, and B2, respectively (**Table 3**). The incidence of severe unsolicited AEs at least possibly or probably related to vaccination was 1% in Groups A1 and B1 and 0% in Groups A2 and B2.

The most frequently reported AEs after any vaccination were in the “general disorders and administration site conditions” system organ class, followed by “infections and infestations”. Overall, the most frequent AE was injection site erythema, with incidences ranging from 37% (Group B1) to 61% (Group A2) of participants in all groups. No AEs leading to withdrawal from the study were reported.

SAEs were reported in 3%–6% of participants; none of them were considered related to vaccination (**Table 3**). All SAEs except 1 (HIV infection in an adult in Group A1) were recovered/resolved by the end of the study. No deaths were reported during the study.

## Discussion

The study evaluated the non-inferiority of the shortened PCECV ID PEP regimen to the recommended 2-sites TRC regimen, in terms of RVNA concentrations ≥0.5 IU/mL at D49, i.e. 42 days after the completion of the 4-site/1-week ID schedule, and 21 days after the completion of the 2-site/TRC ID regimen. The pre-established non-inferiority criterion was successfully met, indicating that the immune response induced by the shortened 4-site/1-week ID regimen is non-inferior to that induced by the currently recommended ID PEP regimen for rabies.

Following both regimens, all participants in all groups achieved RVNA concentrations ≥0.5 IU/mL by D14, considered by the WHO as a marker of an adequate immune response after PEP [1]. This finding is in line with previous observations made for the 2-site/TRC [16] and 4-site/1-week [15] ID regimens. The vast majority of participants in our study maintained adequate RVNA concentrations at approximately 1 year after the completion of the full vaccination course.

Significantly higher RVNA GMCs were observed at D14 in participants having received PCECV according to the 4-site/1-week ID regimen than the 2-site/TRC regimen, similarly to reports from a trial evaluating the immunogenicity of the purified vero cell rabies vaccine (PVRV), following the shortened ID schedule and compared to the TRC regimen [8].However, in our study, non-inferiority of the 4-site/1-week to the 2-site/TRC regimen in terms of RVNA GMC ratio at D49 was not demonstrated. This was probably due to the fact that the time elapsed between the last vaccination and the assessment was considerably longer for the 4-site/1-week ID regimen (42 days) than for the 2-site/TRC 1 month ID schedule (21 days). Of note, significantly higher RVNA GMCs were observed by D7 in the groups receiving the 4-site/1-week ID regimen, even in the absence of HRIG administration. However, the percentages of participants achieving adequate RVNA concentrations ≥0.5 IU/mL—although higher in the 4-site/1-week ID regimen groups than the 2-site/TRC groups—were ≤21% in all groups, suggesting that the shortened regimen does not induce an early onset of protection against rabies. As expected, RVNA concentrations declined after D14 regardless of the used regimen, confirming the trend observed in other 2 clinical trials evaluating the immunogenicity of the 4-site/1-week ID regimen of PCECV and PVRV, with a peak of the immune responses after 2 weeks from the first vaccine doses and a progressive decline in GMCs over time [7, 15].

The trend for higher RVNA GMCs observed in groups receiving the 2-site/TRC ID regimen up to D90 when compared with the 4-site/1-week ID regimen might be once more explained by the difference in days between the last vaccine administration and the time of the assessment. Adequate RVNA levels persisted up to 1 year after the first vaccination in around 90% of participants receiving the 4-site/1-week ID regimen. This percentage was greater than the one observed with the same shortened ID regimen in a previous trial [15] following PCECV and PVRV administration in healthy volunteers (78.9% and 62.5% of participants with RVNA concentrations ≥0.5 IU/mL, respectively) and overall lower than that observed in another clinical study in individuals exposed to rabid animals, where 100% of the evaluated participants had adequate RVNA concentration from D14 up to D365, irrespectively of the rabies vaccine (PCECV or PVRV) received [7]. Moreover, at 1 year post-dose 1, RVNA GMCs were higher in participants having received the shortened ID regimen compared to those having received the recommended TRC course. This finding can be, at least in part, explained by the higher number of PCECV doses administered according to the 4-site/1-week ID regimen compared to the 2-site/TRC ID regimen (overall 12 versus 8, respectively).

Among adults receiving the 4-site/1-week ID regimen, RVNA GMCs and percentages of individuals with RVNA concentrations ≥0.5 IU/mL were consistently higher in the group with no HRIG administration, with differences being statistically significant from D14 to D90. A tendency towards lower RVNA levels when rabies vaccines and HRIG are used concomitantly, compared to administration of the vaccine only has already been reported in the literature [17, 18], but has not been considered clinically relevant. In fact, per WHO [1] and Advisory Committee on Immunization Practice [19] guidance, HRIG administration is part of the recommended management of patients with category III rabid exposure, as it affords protection in the early post-exposure period (D0–D14), when adequate antibody levels induced by vaccination might not yet be mounted [20].

The incidence of AEs and SAEs across all groups was in line with that observed in other studies assessing ID regimens of PCECV [15–17, 21] and did not differ considerably between groups receiving different regimens. The reactogenicity and safety data collected in the pediatric population in our study is supportive of the use of the new ID schedule in this age group, as part of rabies PEP. The 4-site/1-week ID regimen was well-tolerated in the entire study population.

Several efforts have been made over the years to simplify rabies PEP regimens and make them more convenient, including the progressive reduction of the number of doses and visits of the popular Essen IM regimen from 6 doses received over 90 days to an overall 4 doses for healthy individuals (one IM dose on D0, D3, D7 and D14) [19], the adoption of the alternative Zagreb IM regimen (2 doses on D0 [one in each of the 2 deltoid or thigh sites], followed by 1 dose on D7 and D21) and the paradigm shift from IM to ID schedules, with the adoption of the cost-effective 2-site/TRC regimen, especially in low income areas like Africa and Asia where the disease is endemic [3]. This ID regimen still requires a total of 4 clinic visits and approximately 1 month to complete.

With the aim to further simplify rabies PEP, a 4-site/1-week ID regimen–shortening the number of visits to 3 and the duration of the treatment to 1 week–has been developed as an alternative regimen, with the additional expectation to reduce direct and indirect costs associated with clinic visits, and potentially improve compliance to treatment of populations like travelers or those at more remote sites who are in need to be treated within the shortest possible time after contact with rabid animals, due to logistic and economic factors [6].

In our study, the increased number of vaccinations at the same visit did not seem impact compliance to the vaccination schedule, as comparable compliance rates were observed among all groups. Moreover, administering 4 ID doses at the same vaccination visit (accounting for almost half the volume of the reconstituted PCECV) results in less vaccine wastage than when 2 ID doses are administered.

Noteworthy, the study provided data supporting the immunogenicity of the shortened 4-site/1-week ID regimen for PEP in toddlers and children, a population at high risk of contact with rabid animals in countries where the disease is endemic, with persistence data up to 1 year following the PEP start. There are some potential limitations to this trial. First, seeing this was a simulated PEP study, it was conducted in healthy volunteers. Second, RIG was not administered to study participants younger than 18 years due to ethical considerations.

### Conclusions

The administration of PCECV according to a 4-site/1-week ID regimen for rabies PEP was non-inferior to that according to a 2-site/TRC ID regimen in terms of percentages of participants with RVNA concentrations ≥0.5 IU/mL at D49. The elicited immune responses peaked at D14, and subsequently declined up to D365.

RVNA concentrations were consistently lower in individuals with concomitant HRIG administration compared to adults not receiving HRIG, confirming previous observations. However, this was not considered to be clinically relevant, as HRIG administration is part of the recommended PEP procedures. Overall, both PEP regimens were well tolerated.

If included in national and supra-national recommendations for rabies PEP, the 4-site/1-week ID regimen might be a valid alternative to currently recommended 2-site/TRC ID PEP regimen.

## Supporting information

S1 Text Exclusion criteria for enrolment in the study. (DOCX)

S2 Text Note on the trial protocol. (DOCX)

S1 Table Number and percentages of participants with solicited adverse events after any vaccination, and unsolicited and serious adverse events throughout the study, by age stratum (safety set). (DOCX)
