# Supplementary material for: Shortening intradermal rabies post-exposure prophylaxis regimens to 1 week: Results from a phase III clinical trial in children, adolescents and adults
Source: PLoS Negl Trop Dis. 2018 Jun 6;12(6):e0006340. doi: 10.1371/journal.pntd.0006340 (PMC6005579; doi:10.1371/journal.pntd.0006340)
Supplement: S1 Text — (DOCX) [file pntd.0006340.s001.docx]

**S1 Text.** Exclusion criteria for enrolment in the study

Exclusion criteria were:

- prior receipt of rabies vaccine or human rabies immunoglobulin (HRIG)
- prior exposure to rabies
- behavioral or cognitive impairment or psychiatric disease;
- history of or ongoing illnesses which may pose additional risk to the individual;
- any prior or current malignancy for individuals ≥1 to ≤17 years of age;
- malignancy or lymphoproliferative disorder in the 5 years prior to enrolment for individuals ≥18 years of age;
- known or suspected impairment of the immune system;
- females of childbearing potential who had not used any acceptable contraceptive methods for at least 2 months prior to study start or refusal to use such methods up to day 49 of the study;
- pregnant women;
- history of allergy or contraindications to study vaccine or HRIG components;
- receipt or planned receipt of anti-malaria medications;
- concomitant participation in another clinical study (from 30 days prior to first study visit to study end);
- receipt or planned receipt of inactivated or activated vaccines, within 14 days or 28 days from study vaccine administration, respectively;
- body temperature ≥38.0°C (≥100.4°F) within 3 days of intended study vaccination;
- history of drug or alcohol abuse in the 2 years prior to study start.
