# Supplementary material for: Shortening intradermal rabies post-exposure prophylaxis regimens to 1 week: Results from a phase III clinical trial in children, adolescents and adults
Source: PLoS Negl Trop Dis. 2018 Jun 6;12(6):e0006340. doi: 10.1371/journal.pntd.0006340 (PMC6005579; doi:10.1371/journal.pntd.0006340)
Supplement: S2 Text — (DOCX) [file pntd.0006340.s002.docx]

**S2 Text. Note on the trial protocol**

As per convention, the first day of vaccination in the study protocol represents study day 1 (visit 1). To ensure alignment with WHO standards, study days 1, 4, 8, and 29 in this protocol were equivalent to WHO vaccination days 0, 3, 7 and 28 and in this paper have been aligned with the WHO naming convention.
