# Supplementary material for: Shortening intradermal rabies post-exposure prophylaxis regimens to 1 week: Results from a phase III clinical trial in children, adolescents and adults
Source: PLoS Negl Trop Dis. 2018 Jun 6;12(6):e0006340. doi: 10.1371/journal.pntd.0006340 (PMC6005579; doi:10.1371/journal.pntd.0006340)
Supplement: S1 Table — (DOCX) [file pntd.0006340.s003.docx]

# S1 Table. Number and percentages of participants with solicited adverse events after any vaccination, and unsolicited and serious adverse events throughout the study, by age stratum (safety set)

|  | 1–5 years | |  | 6–17 years | | |  | ≥18 years | | | |
| --- | --- | --- | --- | --- | --- | --- | --- | --- | --- | --- | --- |
|  | Group A1 | Group B1 |  | Group A1 | Group B1 | Group B2 |  | Group A1 | Group A2 | Group B1 | Group B2 |
|  | N=72 | N=71 |  | N=106 | N=107 | N=1* |  | N=178 | N=85 | N=175 | N=88 |
| Any solicited AE | 56 (78%) | 56 (79%) |  | 53 (50%) | 53 (50%) | 1 (100%) |  | 93 (52%) | 55 (65%) | 99 (57%) | 54 (61%) |
| Local | 55 (76%) | 48 (68%) |  | 33 (31%) | 35 (33%) | 1 (100%) |  | 56 (31%) | 35 (41%) | 72 (41%) | 32 (36%) |
| Systemic | 18 (25%) | 19 (27%) |  | 32 (30%) | 31 (29%) | 0 (0%) |  | 69 (39%) | 36 (42%) | 68 (39%) | 41 (47%) |
| Any unsolicited AE | 45 (63%) | 53 (75%) |  | 58 (55%) | 71 (66%) | 1 (100%) |  | 157 (88%) | 71 (84%) | 156 (89%) | 72 (82%) |
| Possibly or probably related | 35 (49%) | 25 (35%) |  | 50 (47%) | 56 (52%) | 1 (100%) |  | 151 (85%) | 71 (84%) | 148 (85%) | 70 (80%) |
| Any SAE | 3 (4%) | 4 (6%) |  | 1 (1%) | 3 (3%) | 0 (0%) |  | 8 (4%) | 3 (4%) | 7 (4%) | 5 (6%) |
| Possibly or probably related | 0 (0%) | 0 (0%) |  | 0 (0%) | 0 (0%) | 0 (0%) |  | 0 (0%) | 0 (0%) | 0 (0%) | 0 (0%) |

*Group A1, participants receiving PCECV according to the 4-site/1-week ID regimen; Group A2, participants receiving PCECV according to the 4-site/1-week ID regimen and HRIG at first visit; Group B1, participants receiving PCECV according to the 2-site/TRC ID regimen; Group B2, participants receiving PCECV according to the 2-site/TRC ID regimen and HRIG at first visit; PCECV, purified chick embryo cell culture vaccine; ID, intradermal; TRC, Thai Red Cross; HRIG, human rabies immunoglobulin; N, number of participants with available results; AE, adverse event; SAE, serious adverse event.*

Note: * One 11-year-old participant was erroneously randomized in Group B2.
